# Inter-subject variation in partition coefficient is largely due to variation in LGE blood T1

**DOI:** 10.1186/1532-429X-15-S1-P48

**Published:** 2013-01-30

**Authors:** Kyung P Hong, Eugene Kholmovski, Sathya Vijayakumar, Derek J Dosdall, Christopher McGann, Ravi Ranjan, Nassir F Marrouche, Daniel Kim

**Affiliations:** 1 Radiology, University of Utah, Salt Lake City, UT, USA; 2 CARMA, University of Utah, Salt Lake City, UT, USA; 3 Bioengineering, University of Utah, Salt Lake City, UT, USA; 4 Internal Medicine, University of Utah, Salt Lake City, UT, USA

## Background

MRI is the only non-invasive modality capable of quantifying diffuse cardiac fibrosis using late gadolinium enhanced (LGE) cardiac T1 mapping. This pulse sequence measures LGE cardiac T1, which directly correlates with the amount of extracellular contrast agent occupying the expanded extracellular matrix containing the interstitial fibrosis, and obviates the need for a reference normal tissue. A practical limitation of the LGE cardiac T1 mapping pulse sequence is that it is sensitive to contrast agent dosage and specific delayed imaging time (i.e., two factors that affect the amount of contrast agent present in tissue). Recently, a method was proposed to address this limitation by combining blood and myocardial T1 measurements pre- and post-contrast agent administration to calculate an index called partition coefficient (λ). However, post-contrast blood T1 can vary across subjects, due to variations in hydration and renal function. We sought to evaluate inter-subject variation in blood T1, myocardial T1, and λ.

## Methods

Six mongrel dogs were imaged under anesthesia at 3T. For LGE T1 mapping, we developed a new cardiac T1 mapping pulse sequence based on B1-insensitive saturation recovery (SR) magnetization pre-conditioning and two single-shot balanced steady-state free precession (b-SSFP) image acquisitions (proton density and T1-weighted) with centric k-space ordering. We performed T1 mapping at baseline and 15 min post contrast agent (0.15 mmol/kg of Gd-BOPTA) administration. We calculated blood T1 and myocardial T1 from our images acquired at baseline and post contrast. The corresponding λ was calculated as: λ =ΔR1,myocardium / ΔR1,blood, where Δ is difference between post contrast and pre contrast, and R1 = 1/T1. We calculated the mean and coefficient of variation (CV) of blood T1, myocardial T1, and λ over 5 dogs.

## Results

**Figure 1:**
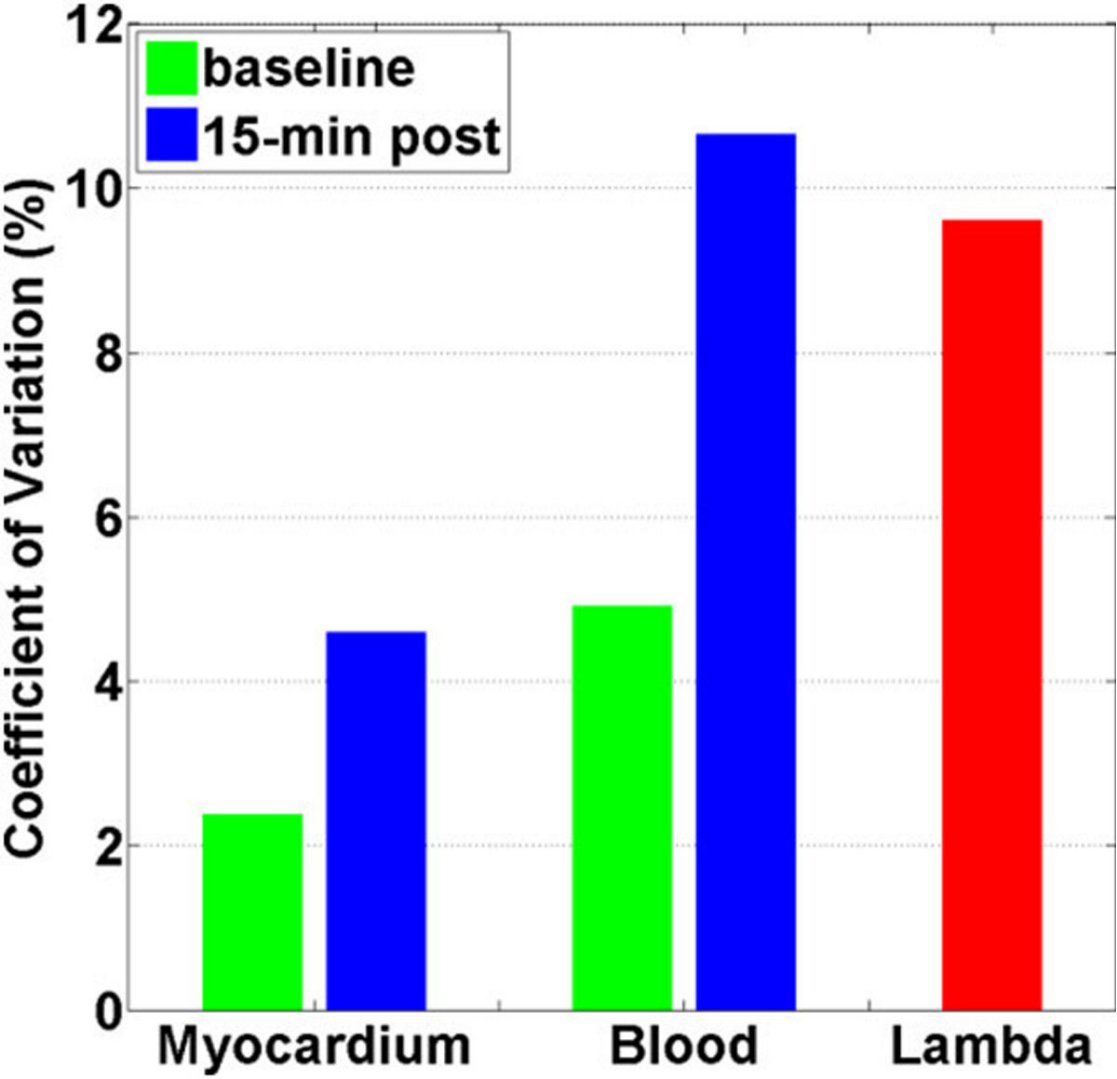
CV of blood T1, myocardial T1, and λ.

Mean blood T1 and myocardial T1 values are summarized in Table 1. The resulting mean λ was 0.35 ± 0.03. Compared with myocardial T1, blood T1 had higher CV at baseline and post contrast (Figure 1). CV of blood T1 at post contrast and λ was 10.7% and 9.6%, respectively.

**Table 1:** Mean blood T1 and myocardial T1.

Tissue type	Baseline	LGE (15 min)
Myocardium	1388 ± 33	935 ± 43
Blood	1757 ± 86	641 ± 68

## Conclusions

We observed that post-contrast blood T1 has high inter-subject variation (~ 10%), likely due to inter-subject variation in hydration and renal function. CV of λ was twice as large as CV of post-contrast myocardial T1. λ yields high inter-subject variation largely due to variation in LGE blood T1.

## Funding

American Heart Association: 0730143N; Ben B. and Iris M. Margolis Foundation

